# Vasodilation and Blood Pressure-Lowering Effect of 3-Demethyl-2-Geranyl-4-Prenylbellidifoline, a Xanthone Obtained from *Garcinia achachairu*, in Hypertensive Rats

**DOI:** 10.3390/plants13040528

**Published:** 2024-02-15

**Authors:** Luísa Nathália Bolda Mariano, Rita de Cássia Vilhena da Silva, Rivaldo Niero, Valdir Cechinel Filho, José Eduardo da Silva-Santos, Priscila de Souza

**Affiliations:** 1 Laboratory of Cardiovascular Biology, Department of Pharmacology, Universidade Federal de Santa Catarina (UFSC), Florianópolis 88040-900, SC, Brazil; luisabolda@univali.br (L.N.B.M.); j.e.silva.santos@ufsc.br (J.E.d.S.-S.); 2 Postgraduate Program in Pharmaceutical Sciences, Núcleo de Investigações Químico-Farmacêuticas (NIQFAR), Universidade do Vale do Itajaí (UNIVALI), Rua Uruguai, 458, Centro, Itajaí 88302-901, SC, Brazil

**Keywords:** bioactive, hypertension, spontaneously hypertensive rats, vasculature, xanthone

## Abstract

3-demethyl-2-geranyl-4-prenylbellidifoline (DGP), a natural xanthone isolated from *Garcinia achachairu*, has previously demonstrated remarkable diuretic and renal protective actions. The present study expands its actions on the cardiovascular system by evaluating its vasorelaxant and blood pressure-lowering effects in spontaneously hypertensive rats (SHRs). Aortic endothelium-intact (E+) preparations of SHRs pre-contracted by phenylephrine and exposed to cumulative concentrations of *G. achachairu* extract, fractions, and DGP exhibited a significant relaxation compared to vehicle-only exposed rings. The non-selective muscarinic receptor antagonist (atropine), the non-selective inhibitor of nitric oxide synthase (L-NAME), as well as the inhibitor of soluble guanylate cyclase (ODQ) altogether avoided DGP-induced relaxation. Tetraethylammonium (small conductance Ca^2+^-activated K^+^ channel blocker), 4-aminopyridine (a voltage-dependent K^+^ channel blocker), and barium chloride (an influx-rectifying K^+^ channel blocker) significantly reduced DGP capacity to induce relaxation without the interference of glibenclamide (an ATP-sensitive inward rectifier 6.1 and 6.2 K^+^ channel blocker). Additionally, administration of DGP, 1 mg/kg i.v., decreased the mean, systolic, and diastolic arterial pressures, and the heart rate of SHRs. The natural xanthone DGP showed promising potential as an endothelium-dependent vasorelaxant, operating through the nitric oxide pathway and potassium channels, ultimately significantly reducing blood pressure in hypertensive rats.

## 1. Introduction

Cardiovascular disease is the leading cause of death in Brazil and worldwide [1,2]. Among cardiovascular disorders, high blood pressure can be highlighted, mainly due to its high prevalence and its role as a risk factor for the development of worsened conditions, such as heart failure and stroke [3]. According to the 7th Brazilian Guidelines for the management of hypertension, hypertension is reached when systolic and diastolic blood pressure are equal to or higher than 140/90 mmHg, respectively [4].

The treatment for hypertension includes drug therapy and lifestyle changes [2,4]. Medications employed for the treatment of hypertension play a pivotal role in managing elevated blood pressure. These medications encompass various classes, each targeting specific physiological pathways to achieve blood pressure control. Examples include angiotensin-converting enzyme (ACE) inhibitors, angiotensin II receptor blockers (ARBs), beta-blockers, diuretics, calcium channel blockers, and others. The choice of medication often depends on individual patient characteristics, comorbidities, and potential side effects. The goal of antihypertensive therapy is not only to lower blood pressure but also to reduce the risk of cardiovascular events and improve overall cardiovascular health. Regular monitoring and adjustments are integral to optimizing treatment outcomes. Notably, antihypertensive medications are effective in managing hypertension for most patients, but these drugs can lead to side effects such as dizziness, fatigue, and electrolyte imbalances, which interfere with patient well-being, reducing adherence to treatment [5].

Therefore, it is worth highlighting the use of medicinal plants, which have a long history in traditional medicine. These natural remedies are valued for their potential to treat various ailments, often with fewer side effects than synthetic drugs. Research continues to uncover their therapeutic benefits, enhancing their role in modern healthcare [6,7]. Many of them are popularly used to prevent and treat cardiovascular disorders, and their bioactives are too [5,8]. In fact, the bioactive compounds produced by plants as a response to environmental challenges often exhibit remarkable therapeutic potential. Secondary metabolites, such as alkaloids, flavonoids, terpenoids, and phenolic compounds, contribute to a plants’ medicinal properties and play a crucial role in human health. These compounds have been harnessed for the development of pharmaceuticals, serving as the basis for numerous drugs used in modern medicine. Exploring the intricate chemistry of plant secondary metabolites not only enriches our understanding of natural remedies but also opens avenues for innovative drug discovery and the treatment of various diseases.

Bioactives from the class of xanthones have shown promising potential in disease treatment [9,10,11,12,13], making them an exciting area of study for developing therapeutic agents. Among these, 3-demethyl-2-geranyl-4-prenylbellidifoline (DGP; or 1,3,5,8-tetrahydroxy-2-geranyl-4-prenylxanthone), a natural xanthone isolated from *Garcinia achachairu*, has previously demonstrated remarkable diuretic and renal protective properties [14]. 

The present study expands its actions on the cardiovascular system by evaluating its vasorelaxant and blood pressure-lowering effects in spontaneously hypertensive rats (SHRs). Examining the vasorelaxant and hypotensive effects of this xanthone, in addition to its previously established diuretic properties, provides a broader assessment of its therapeutic potential against cardiovascular disorders. This study not only enhances our understanding of the pharmacology of DGP but also reinforces this xanthone as a promising candidate able to address a broader range of complications associated with systemic arterial hypertension.

## 2. Results and Discussion

*G. achachairu*, native to South America, particularly in the Amazon rainforest, is a medicinal plant characterized by glossy green leaves and small, yellow, aromatic fruits. Also referred to as “achachairu”, this evergreen tree belongs to the Clusiaceae family and has been traditionally utilized by indigenous communities for its medicinal benefits. Preparations obtained from different parts of this plant have already been the subject of studies revealing antimicrobial [15], gastroprotective [16], anti-inflammatory [17], and diuretic [18] properties. In the present study, we extended these actions to understand the benefits of this plant in mechanisms that contribute to the control of blood pressure, since the demonstration of diuretic activity has already opened this promising perspective. The previous identification of diuretic activity has paved the way for this promising avenue of research. To achieve this objective, we initiated a screening process for biological activity utilizing the crude extract and fractions obtained using solvents with varying polarities, employing the isolated rat aorta methodology. Each solvent possesses specific characteristics that determine its effectiveness in extracting different types of compounds.

**Figure 1:**
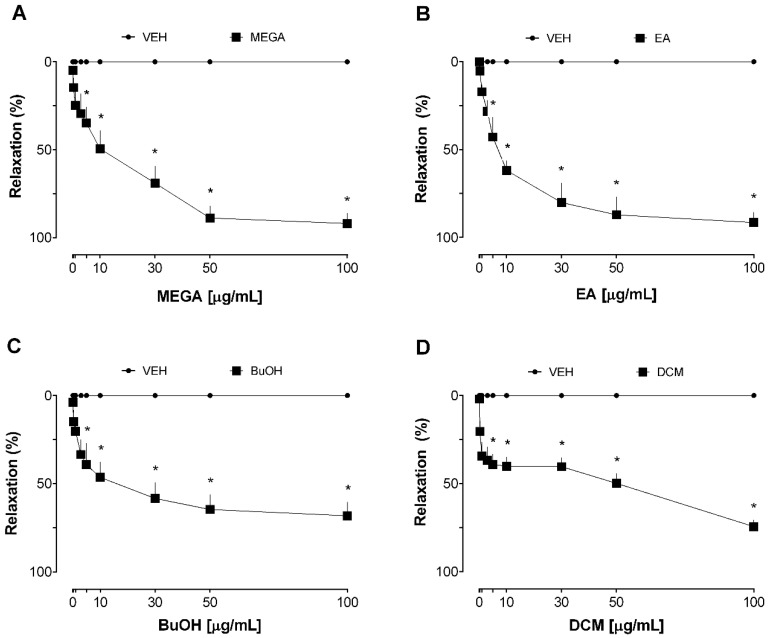
Vasorelaxation induced by extract and fractions obtained from *G. achachairu* branches in the aorta of spontaneously hypertensive rats (SHRs). (**A**) MEGA. (**B**) EA fraction. (**C**) BuOH fraction. (**D**) DCM fraction. Statistical analyses were performed using a two-way analysis of variance followed by Bonferroni’s multiple comparison test. * *p* < 0.05 versus VEH.

Thus, the first experiment was carried out to investigate the vasorelaxant effect of methanolic extract from *G. achachairu* branches (MEGA) in isolated thoracic aorta arteries from SHRs. The isolated rat aorta model offers valuable insights into vascular physiology and pharmacology. This well-established methodology allows for researchers to study the effects of various compounds and physiological conditions on vascular tone. It provides a foundation for understanding vascular function and potential therapeutic interventions in cardiovascular research. The cumulative administration of MEGA resulted in a concentration-dependent vasorelaxant response in the endothelium-intact aortic rings of SHRs (Figure 1A). The maximal relaxation (Rmax) value was ~92%. The VEH group, indicated by the closed circle symbol in all figures, exhibited no discernible impact on the tonus of the aortic rings. In addition, the relaxation induced by both branch-derived fractions were significant when compared with the VEH group. The Rmax values were ~92% for the ethyl acetate (EA) fraction (Figure 1B), ~69% for butanolic (BuOH) fraction (Figure 1C), and ~75% for dichloromethane (DCM) fraction (Figure 1D). The relaxation obtained by acetylcholine was used in all preparations to determine tissue viability and as a positive control for the tests, which reached a relaxation close to 100%.

**Figure 2:**
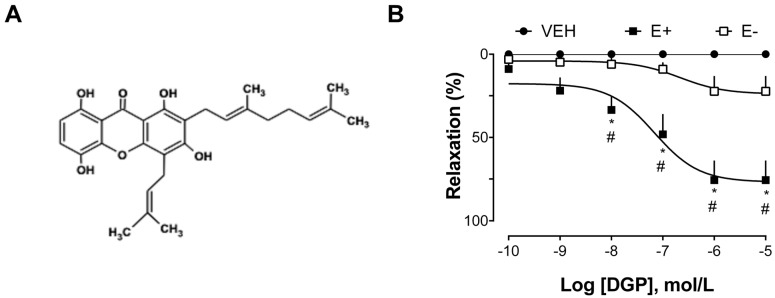
Vasorelaxation induced by DGP in the aorta of spontaneously hypertensive rats (SHRs). (**A**) Chemical structure of DGP: 3-demethyl-2-geranyl-4-prenylbellidypholine xanthone. (**B**) Concentration–response curves were determined in endothelium-intact (E+) or endothelium-denuded (E−) aortic rings. Statistical analyses were performed using a two-way analysis of variance followed by Bonferroni’s multiple comparison test. * *p* < 0.05 versus VEH. # *p* < 0.05. versus other groups. VEH: vehicle.

The next group of experiments was carried out to explore the vasorelaxant effect of xanthone DGP in isolated thoracic aorta arteries from SHRs. As shown in Figure 2B, DGP induced a significant relaxation response in the aortic rings of SHRs, with a maximum effect of 75.54 ± 28.51% in endothelium-intact, and 22.26 ± 20.47% in endothelium-denuded rings. SHR animals are advantageous for studying the mechanisms involved in endothelial dysfunction conditioned by hypertensive vasoconstriction [19]. Furthermore, the hypertension found in SHR animals is similar to hypertension in humans [20], so they are commonly used as an experimental model to study the underlying mechanisms of hypertension and to assess the effectiveness of therapeutic interventions. Indeed, the physiopathological changes found in SHRs include an increase in systolic blood pressure, endothelial dysfunction, vascular remodeling, cardiac hypertrophy, alterations in vascular reactivity, and increased arterial stiffness, among others. These changes are characteristic of the hypertensive state and are often associated with cardiovascular complications.

**Figure 3:**
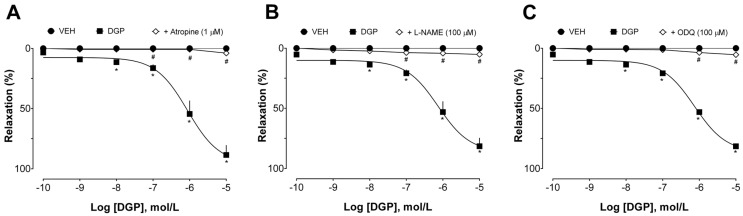
Participation of the muscarinic receptor and nitric oxide pathway in DGP-induced relaxation in spontaneously hypertensive rats (SHRs) aortic rings. (**A**) Effect of DGP in the absence or presence of atropine; (**B**) effect of DGP in the absence or presence of L-NAME; and (**C**) effect of DGP in the absence or presence of ODQ. Statistical analyses were performed using a two-way analysis of variance followed by Bonferroni’s multiple comparison test. * *p* < 0.05 versus VEH. # *p* < 0.05. versus other groups. DGP: 3-demethyl-2-geranyl-4-prenylbellidypholine xanthone. VEH: vehicle.

Additional experiments were conducted to investigate the mechanisms involved in the vasorelaxant effect induced by DGP in endothelium-intact aortic rings from SHRs. First, the effect of DGP was evaluated in the presence of atropine (non-selective muscarinic receptor antagonist), L-NAME (non-selective nitric oxide synthase inhibitor), and ODQ (inhibitor of soluble guanylate cyclase). As can be seen in Figure 3A, atropine suppressed DGP-induced relaxation (Emax 3.87 ± 1.88%). These data suggest that the vasorelaxant effect of DGP depends, at least in part, on the activation of muscarinic receptors. Muscarinic acetylcholine receptors play a pivotal role in vascular physiology. Activation of the M3 subtype of muscarinic receptors in arteries is a key event in modulating endothelial activity and vascular tone. The stimulation of these receptors triggers a series of intracellular responses that lead to vasodilation [21,22]. This effect is characterized by the relaxation of smooth muscle cells in the arterial walls [23], resulting in increased blood flow and decreased vascular resistance, which can have important implications for cardiovascular biology and blood pressure regulation [24]. Notably, Mariano et al. [14] have previously evaluated the possible mode of diuretic action of DGP, demonstrating that pretreatment with atropine prevented the diuretic effect of DGP, corroborating the findings described in this study.

As shown in Figure 3B,C, L-NAME and ODQ also prevented DGP-induced relaxation (Emax 5.13 ± 2.75% and 5.39 ± 3.26%, respectively). These data suggest that the vasorelaxant effects of DGP are widely dependent on the nitric oxide (NO) pathway. Briefly, NO is an endothelium-derived vasorelaxant substance that participates in the control of basal vascular tone, vascular resistance, and blood pressure regulation [25]. NO is formed from L-arginine metabolism by three isoforms of NO synthase (NOS) in response to various stimuli [26]. NO synthesized by endothelial NOS has been considered the most abundant and important endothelial mediator for regulating vasoreactivity [27]. NO is released from the endothelium and spreads quickly to smooth muscle cells, activating the soluble guanylate cyclase (sGC). The increased intracellular levels of cyclic guanosine monophosphate (cGMP) are responsible for inducing vasorelaxation [28,29]. Thus, it is reasonable to state that the vasodilatory effects found in this study are a consequence of activation of the NO/cGMP pathway, since L-NAME, a NOS inhibitor, and ODQ, a sGC inhibitor, significantly inhibited the vascular relaxation induced by DGP.

The role of adrenergic receptors was also investigated in an attempt to further explore the vasorelaxant effect of DGP. Adrenergic receptors are fundamental for vascular biology. Endogenous catecholamines such as epinephrine and norepinephrine are natural ligands of these receptors. Depending on the receptor subtype involved, activation of adrenergic receptors can lead to either vasoconstriction or vasodilation. Alpha-1 adrenergic receptors are typically involved in vasoconstriction, leading to a narrowing of blood vessels [30]. In contrast, stimulation of beta-adrenergic receptors often leads to vasodilation, promoting the relaxation of blood vessels [31]. In our experiments, the beta-blocker propranolol did not change the DGP-induced vasodilation, indicating that activation of beta-adrenergic receptors is not involved in the vascular effects of DGP.

Several endothelial mediators act by producing vasoconstriction and vasodilation, contributing to the maintenance of the vascular tone. Among these, cyclooxygenase-derived prostanoids significantly influence vascular physiology. The balance and dynamic interplay of prostanoids in blood vessels contribute to regulating the vascular tone, controlling blood pressure, blood flow, and cardiovascular homeostasis [32]. Importantly, the incubation of indomethacin, a well-stablished cyclooxygenase inhibitor, was unable to modify DGP-mediated relaxation in Phe-contracted aortic rings, suggesting that these endothelial-derived prostanoids do not contribute to the vascular effects of this xanthone.

Additionally, the involvement of K^+^ channels in the vasorelaxant effect of DGP in the aortas of SHRs was also investigated. Potassium channels play a pivotal role in arterial relaxation. Activation of these channels leads to the efflux of potassium ions from vascular smooth muscle cells, hyperpolarizing the cell membrane and promoting vasorelaxation. This process results in the relaxation of arterial walls, increased blood vessel diameter, reduced vascular tone, and improved blood flow [33]. There are various subtypes of potassium channels in blood vessels, each one with specific roles in vascular physiology. These channels can be broadly categorized into calcium- and sodium-activated (K_Ca,_ K_Na_, respectively), voltage-gated (K_V_), and inwardly rectifying (K_IR_) potassium channels, and two-pore domain (K_2P_) potassium channels. The activation of these channels is highly regulated. Briefly, the activation of potassium channels on the cellular membrane of smooth muscle cells within arteries results in an outflow of K^+^, leading to hyperpolarization of the membrane potential. This action subsequently shuts down voltage-dependent calcium (Ca^2+^) channels, reducing Ca^2+^ influx, ultimately inducing vasodilation.

**Figure 4:**
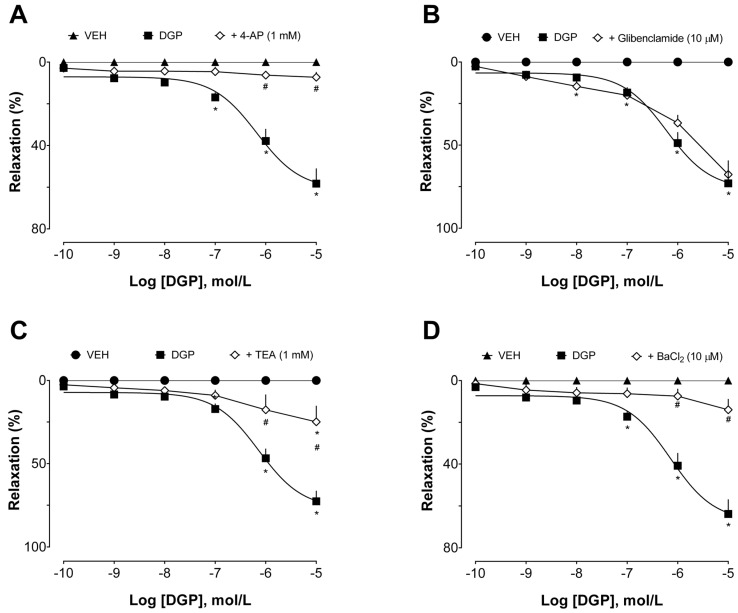
Influence of K^+^ channels on the vasorelaxant effect of DGP. (**A**) Effect of DGP in the absence or presence of 4-AP; (**B**) effect of DGP in the absence or presence of glibenclamide; (**C**) effect of DGP in the absence or presence of TEA; (**D**) effect of DGP in the absence or presence of BaCl2. Statistical analyses were performed using a two-way analysis of variance followed by Bonferroni’s multiple comparison test. * *p* < 0.05 versus VEH. # *p* < 0.05 versus other groups. DGP: 3-demethyl-2-geranyl-4-prenylbellidypholine xanthone. VEH: vehicle. 4-AP: 4-aminopyridine. TEA: tetraethylammonium. BaCl2: barium chloride.

The intricate interplay of these potassium channel subtypes is essential for precisely controlling vascular tone and, consequently, blood pressure regulation [34]. We used pharmacological tools with selective effects on these channels [35] to investigate their involvement in DGP-induced vasorelaxation (Figure 4). Tetraethylammonium (in a concentration range to selectively inhibit K_Ca_ subtypes 2.1, 2.2, and 2.3, also known as small-conductance calcium-activated potassium channels), 4-aminopyridine (a selective K_V_ blocker), and barium chloride (a non-selective K_IR_ blocker) significantly reduced DGP capacity to induce relaxation, which was not sensitive to glibenclamide (a selective K_ir_ 6.1 and 6.2 blocker, also known as ATP-sensitive inward rectifier potassium channels). Together, this set of experiments suggests that potassium channels, especially small-conductance calcium-activated, voltage-gated, and inwardly rectifying potassium channels, but not ATP-sensitive potassium channels, are pivotal elements in the downstream cascade of events mediating the vasorelaxant effects induced by DGP.

**Figure 5:**
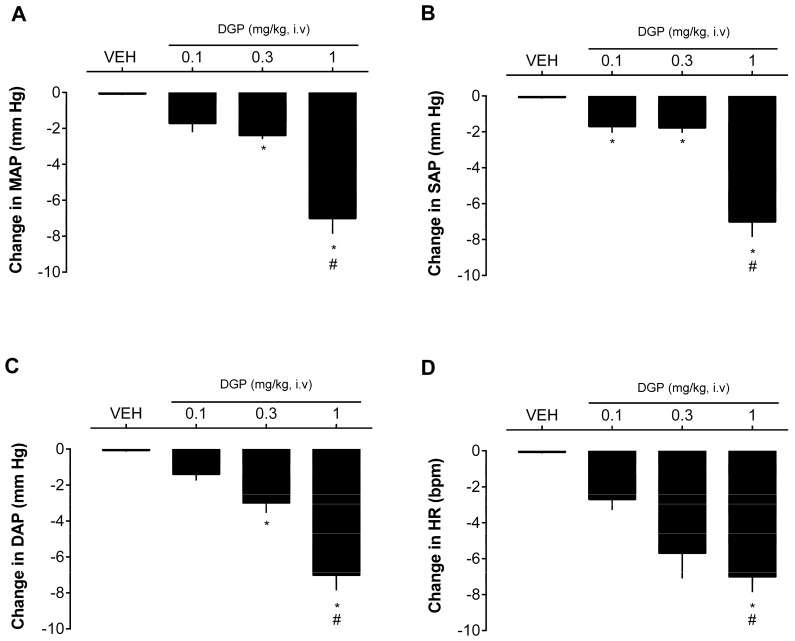
Blood pressure-lowering effect of DGP in spontaneously hypertensive rats (SHRs). Statistical analyses were performed using a one-way analysis of variance followed by Bonferroni’s multiple comparison test. * *p* < 0.05 versus VEH. # *p* < 0.05 versus 0.1 mg/kg group. DGP: 3-demethyl-2-geranyl-4-prenylbellidypholine xanthone. VEH: vehicle. (**A**) MAP: mean arterial pressure. (**B**) SAP: systolic arterial pressure. (**C**) DAP: diastolic arterial pressure. (**D**) HR: heart rate.

Finally, the effect of DGP on the blood pressure values of SHRs was also evaluated. In this experiment, acetylcholine was also used as a positive control for the tests due to its potent hypotensive action. Figure 5 shows that intravenous injection of DGP caused a dose-dependent decrease in blood pressure at doses of 0.3 and 1 mg/kg. The drop generated by the highest dose administered reached 7.02 ± 0.84 mmHg in MAP (Figure 5A), 6.49 ± 0.53 mmHg in SAP (Figure 5B), 7.01 ± 1.04 mmHg in DAP (Figure 5C), and 11.02 ± 3.11 bpm in heart rate (Figure 5D). The reduction in blood pressure is of paramount importance in the therapeutic management of hypertension and other hypertension-associated cardiovascular diseases. Lowering blood pressure serves as a cornerstone in mitigating the risk of cardiovascular events, such as heart attacks and strokes. Furthermore, it can alleviate the strain on the heart and blood vessels, ultimately enhancing cardiovascular health. Therefore, achieving and maintaining optimal blood pressure levels is fundamental for the well-being and long-term prognosis of individuals with cardiovascular diseases [36]. Hence, this research suggests a promising avenue for future exploration and potential use of this xanthone in the context of cardiovascular disorders. Nevertheless, while the findings presented here are significant, additional investigations are warranted to gain a more comprehensive understanding on the mechanisms underpinning the vascular (including other vascular beds) effects of DGP and its extended impact on blood pressure over prolonged treatment.

## 3. Materials and Methods

### 3.1. Plant Material

In August 2019, branches from *G. achachairu* were gathered in Camboriú city, Santa Catarina, Brazil. The identification was carried out by Dr. Oscar B. Iza from the University of Vale of Itajai (UNIVALI), Itajai, SC, Brazil. A voucher specimen was officially deposited at the Barbosa Rodrigues Herbarium (Itajai-SC, Brazil) under the reference number HBR 52,637.

The branches of *G. achachairu*, after being dried in a plant drying room with controlled humidity, underwent pulverization individually using knife mills (Ø 0.5 cm). The pulverized material was subjected to static maceration with methanol, maintaining a drug–solvent ratio of 1:20 at room temperature for seven days. Following filtration, the solvent was eliminated by evaporation under reduced pressure in a rotary evaporator with temperature control set at approximately 50 °C, resulting in a concentrated methanolic extract (MEGA) weighing 168 g (10.31% yield). A portion of the MEGA was reconstituted in a methanol–H_2_O mixture (60:40) and underwent liquid–liquid partition using solvents with increasing polarity. This process included three rounds with 300 mL each of dichloromethane (DCM, 37.40 g—22.26% yield), ethyl acetate (EA, 28.30 g—16.84%), and butanol (BuOH, 45.50 g—27.08%), producing the respective semi-purified fractions. All fractions were subsequently concentrated using a rotary evaporator.

### 3.2. Xanthone Isolation

The process of isolating 3-demethyl-2-geranyl-4-prenylbellidifoline (DGP; Figure 2A) from *G. achachairu* branches has been thoroughly documented in a prior publication authored by Mariano et al. [37]. In summary, the methodology encompasses the extraction of 620.29 g of powdered *G. achachairu* branches using methanol at room temperature over a 7-day period. The resulting extract, comprising 6.16% (38.21 g), was subsequently suspended in a methanol–water (60:40) mixture and underwent liquid–liquid partition utilizing dichloromethane, ethyl acetate, and butanol as sequentially polar solvents. A portion of the soluble dichloromethane (5.1 g) underwent column chromatography on silica-gel, employing hexane–acetone in increasing polarity for elution, yielding 70 fractions. Sub-fraction 16–20 (77.97 mg—0.22%) was characterized as 3-demethyl-2-geranyl-4-prenybellidifoline through various analyses, including NMR, DEPT, HMBC, and IR data. This identification was confirmed after elution with hexane–acetone (80:20) in thin layer chromatography and development with ferric chloride.

### 3.3. Drugs

*N*-ω-Nitro-L-arginine methyl ester (L-NAME), phenylephrine hydrochloride, tetraethylammonium (TEA), glibenclamide, 4-aminopyridine (4-AP), 1H-[1,2,4]oxadiazolo[4,3-a]quinoxalin-1-one (ODQ), indomethacin, atropine, propranolol, sodium chloride (NaCl), potassium chloride (KCl), sodium bicarbonate (NaHCO_3_), magnesium sulfate (MgSO_4_), calcium chloride (CaCl_2_), potassium dihydrogen phosphate (KH_2_PO_4_), and glucose were all purchased from Sigma–Aldrich, Inc. (St. Louis, MO, USA).

### 3.4. Animals

Male spontaneous hypertensive rats (SHRs) aged between 3 to 4 months, provided by the University of Vale do Itajaí (UNIVALI, Brazil), were used in this study. The animals were housed in a controlled environment with a room temperature of 22 ± 2 °C, and subjected to a 12 h light/dark cycle. The animals were given free access to both water and Nuvilab Cr-1 commercial feed, supplied by Nuvilab Produtos Agropecuários LTDA (Colombo, Brazil). The nutritional composition of the feed included an energy content of 13,776 kJ/Kg, comprising 530 g/kg of carbohydrates, 220 g/kg of proteins, 40 g/kg of lipids, and 90 g/kg of ash. All experimental protocols and techniques were subjected to review and authorization by UNIVALI’s ethics committee (approval number 013/2020) and were conducted in compliance with all the established ethical guidelines.

### 3.5. Preparation of Aortic Rings and Tension Measurement

The rats were subjected to anesthesia using xylazine (10 mg/kg) and ketamine (80 mg/kg), administered intraperitoneally. The descending thoracic aorta was then extracted, cleaned of its surrounding connective tissue, and cut into 4–5 mm long rings. The aortic rings were affixed to holders attached to transducers and mounted in a glass chamber filled with Krebs nutritive solution composed of the following (in mM): NaCl 115.3, KCl 4.9, NaHCO_3_ 25, MgSO_4_ 1.2, CaCl_2_ 2.49, KH_2_PO_4_ 1.2, and glucose 11.1. The solution was continuously oxygenated with a carbogen mixture (95% O_2_/5% CO_2_) and maintained at 37 °C. The aortic rings were subjected to a baseline tension of 1 g. Isometric transducers connected to DATAQ Instruments data acquisition hardware through a signal amplifier and a computer running the integration software (WinDaq software DI-1100, DATAQ Instruments, Akron, OH, USA) were used to record the vessel tone. An initial stabilization period of 1 h followed, in which the Krebs solution was replaced every 15 min. After this period, the vessels were stimulated by changing the Krebs solution for potassium chloride solution (KCl—60 mM). Subsequently, a 30 min interval was allowed for a new stabilization of the preparations. A second contractile response was obtained by adding 1 μM phenylephrine (Phe), followed by the administration of 1 μM acetylcholine (Ach) to achieve relaxation, which was used to determine the presence or absence of functional endothelium. Vessels with a functional endothelium were identified based on relaxation equal to or exceeding 80%.

### 3.6. Effect of G. Achachairu Preparations and DGP on Vascular Reactivity

After confirming endothelial integrity, as described above, the rings underwent three consecutive washes with PSS, followed by an additional 60 min stabilization period. To assess the potential vasorelaxant effect of MEGA, EA, BuOH, DCM (0.1–100 µg/mL), and DGP (0.1 nM–10 µM) on the aortic rings, the preparations were precontracted with Phe (1 μM), and cumulative concentrations of each of them were added to the bath during the tonic phase of contraction. The results obtained were expressed as the percentage of relaxation (related to the peak of Phe-induced contraction).

Evaluation of membrane receptor and endothelial mediator involvement in DGP-induced relaxation.

Following the aforementioned tissue preparation and confirmation of aortic viability, the study aimed to assess the influence of muscarinic (M3) and β-adrenergic receptors, prostacyclin, nitric oxide, and guanylate cyclase enzyme on DGP-induced relaxation. Various preparations were subjected to a 30 min incubation in the organ bath with atropine (atro, 1 μM, a non-selective muscarinic receptor antagonist), propranolol (prop, 1 μM, a non-selective α-adrenergic receptor antagonist), indomethacin (indo, 10 μM, a non-selective cyclooxygenase enzyme inhibitor), Nω-nitro-L-arginine methyl ester hydrochloride (L-NAME, 100 μM, a non-selective nitric oxide synthase inhibitor), or 1H-[1,2,4]oxadiazolo[4,3-a]quinoxalin-1-one (ODQ, 10 μM, a selective inhibitor of the soluble guanylate cyclase enzyme). With each substance present in a respective preparation, a new contraction was induced by phe, and during the tonic phase of this contraction, cumulative concentrations of DGP (0.1 nM–10 µM) were added. The results obtained were expressed as the percentage of relaxation (related to the peak of Phe-induced contraction).

### 3.7. Assessment of the Contribution of K^+^ Channels to DGP-Induced Vascular Effects

Following stabilization, various aortic ring preparations were subjected to incubation with specific K^+^ channel blockers, including tetraethylammonium (TEA, 1 mM), in a concentration range to selectively inhibit K_Ca_ subtypes 2.1, 2.2, and 2.3 (also known as small-conductance calcium-activated potassium channels); glibenclamide (GLI, 10 μM), a selective K_ir_ 6.1 and 6.2 blocker (also known as ATP-sensitive inward rectifier potassium channels); 4-aminopyridine (4-AP, 1 mM), a selective voltage-gated K^+^ channel blocker; or barium chloride (BaCl_2_, 10 μM), a non-selective influx-rectifying K^+^ channel blocker. Each preparation was exposed to one of these substances. Subsequently, a new contraction was induced by phe, and during the tonic phase of this contraction, cumulative concentrations of DGP (0.1 nM–10 µM) were added. The results obtained were expressed as the percentage of relaxation (related to the peak of Phe-induced contraction).

### 3.8. Direct Blood Pressure Measurement in SHRs

The rats were anesthetized using a ketamine/xylazine combination (80/10 mg/kg). Additional doses were administered at 45 min intervals to maintain the general anesthesia. The surgical procedures commenced only when the animals became completely unresponsive to pinch stimuli on their pelvic limbs, tail, and surgical areas. A tracheal cannula was inserted to guarantee spontaneous breath throughout the experiment. Polyethylene catheters filled with physiological saline solution (NaCl, 0.9%) were used to cannulate one or both femoral veins, which were used for drug administration. To prevent clotting, a heparin bolus (30 IU) was administered immediately after venous access was established.

The left carotid artery was carefully isolated from the vagus nerve for the insertion of a polyethylene catheter connected to a pressure transducer. We used the AECAD 04P recording system running AQCAD 2.3.7 software (Bonther, Ribeirão Preto, Brazil) for continuous measuring of the mean arterial pressure (MAP), systolic arterial pressure (SAP), diastolic arterial pressure (DAP), and heart rate (HR). After 20 min of blood pressure stabilization, different doses of DGP (0.1, 0.3, and 1 mg/kg) were administered intravenously. Each subsequent injection was given only after the effects of the previous one had worn off, or a 10 min interval was respected between the injections. The results are expressed as changes in pressure or HR from the baseline mean immediately prior to DGP administration. The values were compared with the group that received only the vehicle.

### 3.9. Statistical Analysis

The data are presented as the mean ± standard error of the mean (SEM) of six aortic rings (obtained from different animals) or six rats (blood pressure measurement) per groups. Statistical analysis was performed using one- or two-way analysis of variance (ANOVA) followed by Bonferroni’s post hoc test. Statistical significance was defined as *p* < 0.05.

## 4. Conclusions

In conclusion, 3-demethyl-2-geranyl-4-prenylbellidifoline (DGP), a natural xanthone isolated from *G. achachairu*, has demonstrated notable vasorelaxant and blood pressure-lowering effects in SHRs. The vasodilatory action of DGP was evidenced in aortic endothelium-intact preparations of SHRs, where it induced significant relaxation compared to the vehicle-only exposed rings. The involvement of the NO pathway and potassium channels, specifically the small-conductance Ca^2+^-activated K^+^ channels, voltage-dependent K^+^ channels, and influx-rectifying K^+^ channels, were crucial for the observed vasorelaxation. Furthermore, the systemic administration of DGP at a dose of 1 mg/kg intravenously resulted in a substantial reduction in mean, systolic, and diastolic arterial pressures, along with a decrease in heart rate in SHRs. These findings underscore the promising potential of DGP as an endothelium-dependent vasorelaxant and contributor to a significant reduction in blood pressure in hypertensive rats. Nevertheless, additional investigations are warranted to validate the proposed mechanism of action outlined in this study, which implicates the potential involvement of the NO/cGMP and K^+^ channel pathways.

## Data Availability

Data will be made available on reasonable request.

## Abbreviations

3-demethyl-2-geranyl-4-prenybellidifoline (DGP); 4-aminopyridine (4-AP); 1H-[1,2,4]oxadiazolo[4,3-a]quinoxalin-1-one (ODQ); calcium chloride (CaCl_2_); calcium-activated potassium channel (K_Ca_); cyclic guanosine monophosphate (cGMP); inwardly rectifying potassium channel (K_IR_); *N*-ω-Nitro-L-arginine methyl ester (L-NAME); nitric oxide (NO); nitric oxide synthase (NOS); phenylephrine hydrochloride; potassium chloride (KCl); potassium dihydrogen phosphate (KH_2_PO_4_); sodium chloride (NaCl); sodium bicarbonate (NaHCO_3_); sodium-activated potassium channel (K_Na_); soluble guanylate cyclase (sGC); spontaneously hypertensive rats (SHRs); magnesium sulfate (MgSO_4_); tetraethylammonium (TEA); two-pore domain (K_2P_) potassium channels; vehicle (VEH); voltage-gated potassium channel (K_V_).
